# Violence and discrimination among Ugandan residents during the COVID-19 lockdown

**DOI:** 10.1186/s12889-021-10532-2

**Published:** 2021-03-08

**Authors:** Elizabeth Katana, Bob Omoda Amodan, Lilian Bulage, Alex R. Ario, Joseph Nelson Siewe Fodjo, Robert Colebunders, Rhoda K. Wanyenze

**Affiliations:** 1 Uganda Public Health Fellowship Program, Ministry of Health, Kampala, Uganda; 2 Global Health Institute, University of Antwerp, Antwerp, Belgium; 3 School of Public Health, College of Health Sciences, Makerere University, Kampala, Uganda

**Keywords:** COVID-19, Lockdown, Violence, Discrimination, Epidemic, Uganda, Law enforcement, Police violence, Sub-Saharan Africa

## Abstract

**Background:**

In March 2020, the World Health Organization (WHO) declared COVID-19 a pandemic. Many countries in Sub-Saharan Africa, Uganda inclusive, implemented lockdowns, curfew, banning of both private and public transport systems, and mass gatherings to minimize spread. Social control measures for COVID-19 are reported to increase violence and discrimination globally, including in Uganda as some may be difficult to implement resulting in the heavy deployment of law enforcement. Media reports indicated that cases of violence and discrimination had increased in Uganda’s communities following the lockdown. We estimated the incidence and factors associated with experiencing violence and discrimination among Ugandans during the COVID-19 lockdown to inform control and prevention measures.

**Methods:**

In April 2020, we conducted a secondary analysis of cross-sectional data under the International Citizen Project (ICP) to assess adherence to public health measures and their impact on the COVID-19 outbreak in Uganda. We analyzed data on violence and discrimination from the ICP study. We performed descriptive statistics for all the participants’ characteristics and created a binary outcome variable called experiencing violence and/or discrimination. We performed logistic regression analysis to identify the factors associated with experiencing violence and discrimination.

**Results:**

Of the 1726 ICP study participants, 1051 (58.8%) were males, 841 (48.7%) were currently living with a spouse or partner, and 376 (21.8%) had physically attended work for more than 3 days in the past week. Overall, 145 (8.4%) experienced any form of violence and/or discrimination by any perpetrator, and 46 (31.7%) of the 145 reported that it was perpetrated by a law enforcement officer. Factors associated with experiencing violence or discrimination were: being male (AOR = 1.60 CI:1.10–2.33), having attended work physically for more than 3 days in the past week (AOR = 1.52 CI:1.03–2.23), and inability to access social or essential health services since the epidemic started (AOR = 3.10 CI:2.14–4.50).

**Conclusion:**

A substantial proportion of Ugandan residents experienced violence and/or discrimination during the COVID-19 lockdown, mostly perpetrated by law enforcement officers. We recommend mitigation of the collateral impact of lockdowns with interventions that focus on improving policing quality, ensuring continuity of essential services, and strengthening support systems for vulnerable groups including males.

## Background

In March 2020, the World Health Organization (WHO) declared COVID-19 a pandemic [1]. At the time, there were more than 4000 deaths due to COVID-19 and about 118,000 confirmed cases globally, and the disease had reached every continent except Antarctica [2]. The COVID-19 preventive strategies instituted globally included: promotion of the use of masks in public, frequent and proper hand washing and hygiene, and promotion of physical distancing. By April 2020, 43 of the 46 Sub Saharan African countries had reported confirmed cases of COVID-19 – 13 of them, Uganda inclusive, implemented nationwide lockdowns alongside other public health measures, while 10 countries implemented partial lockdowns in hotspots [3]. Some of the key preventive strategies in Uganda’s response to COVID-19 included nationwide curfew from 6.30 am to 7 pm, banning of both private and public transport systems, and mass or social gatherings.

Public health and social control measures for COVID-19 including lockdowns, stay-at-home orders, and physical distancing restrictions are reported to increase violence and/or discrimination globally, including in Uganda [4, 5]. According to reports from several countries, some of these control measures may be difficult to implement through existing community leadership constructs resulting in a backup or heavy deployment of law enforcement officers including the military, which raises the likelihood of police-citizen conflicts [5–7].

Early in the COVID-19 epidemic, there were several media reports of security forces brutalizing and torturing civilians in some of the Sub-Saharan African countries that had instituted lockdowns including South Africa, Nigeria, Uganda, and Kenya with accounts that some of the brutality contradicted with the measures put in place to allow continuity of essential services [8]. Additionally, there was a sharp increase in gender-based violence (GBV) dubbed as the “the shadow epidemic” alongside the COVID-19 epidemic globally including in Sub-Saharan Africa with worries of underreporting, or lack of or access to data potentially underestimating the number of cases [9]. For example, there was a monthly increase of 149% in reports of GBV cases following the introduction of lockdowns at the end of March 2020 in Nigeria, while there was a significant spike in sexual offenses in Kenya in early April 2020 [4, 9].

Violence because of the social measures taken against COVID-19 in this context refers to individuals experiencing any form of intentional use of force or power against them with potential for injury or death by any perpetrators known or unknown to them while in their homes or communities [10]. Discrimination is a public health issue commonly defined as unfair or prejudicial treatment of people or groups of people based on characteristics such as age, race, gender, or sexual orientation [11]. In this context, discrimination refers to individuals experiencing any negativity towards them due to the social measures of COVID-19 resulting from their social or economic status, ethnicity, race, or nationality [12]. In the absence of the COVID-19 epidemic and the social control measures, these specific accounts or experiences of violence and/or discrimination would probably not have occurred. Some studies have shown that accounts of violence and discrimination often happen or exist together especially among vulnerable groups including women and disabled persons hence requiring platforms and interventions that can address violence and discrimination simultaneously [13–15].

According to the 2016 Uganda Demographic and Health Survey (UDHS), violence and discrimination among Ugandans, occurring in combination or isolation from each other in the 12 months preceding the survey was reported as physical violence (20%), sexual violence among women (13%) and men (4%), and spousal violence (39%) for each of the sexes [16]. Potential influencers of violence and/or discrimination included age, sex, employment status, employed vs unemployed, education level, wealth status, rural vs urban settings, having a disability, race or ethnicity, lifestyles such as alcohol consumption, substance use, and marital status among others [16, 17].

The COVID-19 preventive measures implemented in Uganda resulted in citizens spending more time in their respective homesteads, communities, or neighborhoods. Media reports indicated that cases of domestic violence increased in the communities following the implementation of the lockdown [18]. Other forms of violence and discrimination such as brutality by law enforcement officers and discrimination were also reported [19]. In this study, we assessed four forms of violence and/or discrimination including physical violence at home, physical violence outside the home, discrimination because of social/economic status, and discrimination because of ethnicity, race, or nationality. We aimed to estimate the incidence and predictors of violence and discrimination among Ugandan residents during the initial phase (first two months) of the COVID-19 epidemic to inform control and prevention measures during similar epidemics.

## Methods

### Study design and data source

We conducted a secondary analysis of cross-sectional data under the International Citizen Project (ICP) to assess adherence to public health measures and their impact on the COVID-19 outbreak, initiated by an international group of researchers from Asian, African, South American, and European countries. The protocol and questionnaire for the ICP survey are largely based on the citizen science Corona survey first launched in Belgium by the university of Antwerp on March 17, 2020, and adopted by 21 countries globally including Uganda in April 2020 [20]. The ICP project was implemented through a cross-sectional survey design with an online questionnaire that had six modules: socio-demographics, daily and professional life during the COVID-19 lockdown, community and personal preventive measures for COVID-19, and personal health questions [20]. The questionnaire can be accessed via the www.ICPcovid.com website.

The questionnaire was deployed in Uganda on April 16, 2020 (day 22 of Uganda’s total lockdown) and circulated widely via email, WhatsApp, Facebook, and Twitter platforms. The survey collected responses from Ugandan residents nationwide until April 30, 2020.

### Study variables

For this study, we considered data from four domains of the ICP study-Uganda. The first domain asked questions about general socio-demographic information (including age, sex, religion, education, location, marital status, and housing conditions).

Participants were asked questions about their professional life during the COVID-19 epidemic examples included “what are your current working conditions (working from home, in an open space, closed indoor space alone, closed indoor space with several people and unemployed or student)?”, “what transportation means did you use to go to work (public or private means, hired vehicles, or by foot)?”, “how many days did you go to work in the past week?”, and “are you working from home today”?

Participants were also asked questions on their daily life during the COVID-19 epidemic, examples were “during the last week did you have any difficulties obtaining food (yes or no)”? “how many people apart from your housemates did you talk to yesterday face to face”? and “during the last week, on a scale of 5, how worried were you about your health”?

The last domain asked personal health questions, examples were “have you been eating more healthy foods such as fruits and vegetables since the COVID-19 epidemic started (yes or no)”? “did you have flu-like symptoms in the past week (yes, no or don’t know)”? “do you smoke (yes or no)” “do you have an underlying disease (yes or no)”? and “if you have an underlying disease, did you experience any difficulties to obtain your medication since the COVID-19 epidemic started (yes or no)”?

In this study, participants who had experienced difficulties obtaining food and/or essential medicines for underlying conditions were considered unable able to access social services and/or essential health services during the lockdown period.

For the outcome variable, participants were asked questions including “have you suffered any form of violence or discrimination because of the measures taken against the Corona Virus (yes or no)”? “if yes which form? (physical violence at home, physical violence outside the home, discrimination because of my social/economic status or discrimination because of my ethnicity, race or nationality)”? and “who was the perpetrator of this violence or discrimination (family member within the household, other relatives outside the household, other community members who are known to you, other community members unknown to you, law enforcement officer including police, army local defense, etc.)” ?. No specific questions were asked addressing the other common forms of violence including sexual or intimate partner violence, gender-based or emotional violence.

We extracted and cleaned the data using MS Excel 2019 and used STATA 14 for analysis.

### Analysis

We performed descriptive statistical analysis including frequency counts and percentages for all the participants’ characteristics. To identify the factors associated with violence and discrimination, we created a binary outcome variable “experiencing violence or discrimination” integrating any of the four forms of violence and discrimination during the epidemic regardless of the perpetrator. We performed logistic regression analysis to identify the factors associated with experiencing violence and/or discrimination with a significance level of 0.05. We conducted both unadjusted and adjusted logistic regression with the adjusted model only including factors that are significant in the unadjusted model. Discrimination, the goodness of fit, and the degree of deviance explained for the adjusted model were tested through the Hosmer Lemeshow test, the ROC curve, and McFadden’s R-squared respectively.

## Results

### Socio-demographic characteristics of study participants

Data from 1726 ICP study respondents in Uganda were included in the analysis. The mean age of the participants was 36 years, age range 12 to 72 years. Overall, 58.8% (1015/1726) were males, and the majority resided in Kampala city Centre or surrounding suburbs. Of note, almost half of the respondents (779/1726 or 45.1%) reported not being able to access social services such as food and/or essential health services during the lockdown period. Other participant characteristics are summarized in Table 1.

**Table 1:** Socio-demographic characteristics of study participants

Characteristic (***N*** = 1726)	Frequency	Percentage
**Sex**		
Male	1015	58.8
Female	711	41.2
**Age Group**^**a**^		
≤ 17 years	12	0.7
18–28 years	445	25.8
29–39 years	706	40.9
40–49 years	347	20.1
50^+^ years	215	12.5
**Maximum Education**		
Primary & None	3	0.2
Secondary	63	3.7
Tertiary (certificate, diploma, degree)	863	50.0
University (masters & Ph.D.)	797	46.2
**Nationality**		
Ugandans	1679	97.3
Foreigners	47	2.7
**Marital status**		
Single	676	39.2
Legally married	754	43.7
Cohabitation	247	14.3
Divorced & Widowed	49	2.8
**Currently lives with**		
Parent (s)	307	17.8
Spouse/partner	841	48.7
Child (ren)	734	42.5
Sibling (s) or other relative (s)	447	25.9
Friends	115	6.7
Alone	247	14.3
**Lives with housemates in age-groups**		
Over 70 years	179	10.4
Between 18 and 70 years	1495	86.6
12 to 17 years	765	44.3
Under 12 years	1070	62.0
**Lives in:**		
Rural/village	189	11.0
Within Kampala city center	186	10.8
Kampala suburb	688	39.9
Other town/city center	329	19.1
Other suburb	334	19.4
**Housing conditions**		
House or apartment with garden	697	40.4
House or apartment No garden	473	27.4
Apartment with balcony	166	9.6
Room	108	6.3
Apartment No balcony	259	15.0
Hut, Shack & Homeless	23	1.3
**What they do for a living**		
Student	209	12.1
Jobless	124	7.2
Self-employed	284	16.5
Work for a person, institution, or company	731	42.4
Work for the government	378	21.9
**Current working conditions**		
Worker from home	663	38.4
Worker in an open space (market, shop, roadside, etc.)	118	6.8
Worker in a closed indoor space alone (office, etc.)	192	11.1
Worker in a closed indoor space with several others (office, etc.)	300	17.4
Not applicable (jobless or student)	453	26.2
**Days physically attended at work in past week**^**b**^		
0–3 days	1350	78.2
> 3 days	376	21.8
**Wealth Index**		
1st Quintile (poorest)	352	20.4
2nd Quintile	339	19.6
3rd Quintile	368	21.3
4th Quintile	481	27.9
5th Quintile (richest)	186	10.8
**Satisfied with staying at home (on a scale of 5)**		
Not Satisfied (1)	134	7.8
2	133	7.7
3	348	20.2
4	403	23.3
Very Satisfied (5)	708	41.0
**Has difficulty obtaining food**		
Yes	734	42.5
No	992	57.5
**Overall failure to access food and/or essential health services**		
Yes (were not able to access food and/or essential health services)	779	45.1
No	947	54.9

^**a**^ 1 missing value ^b^days physically spent at work category 0–3 days includes those without employment

### Incidence of violence and discrimination among study participants during the COVID-19 lockdown, April 2020

**Fig. 1:**
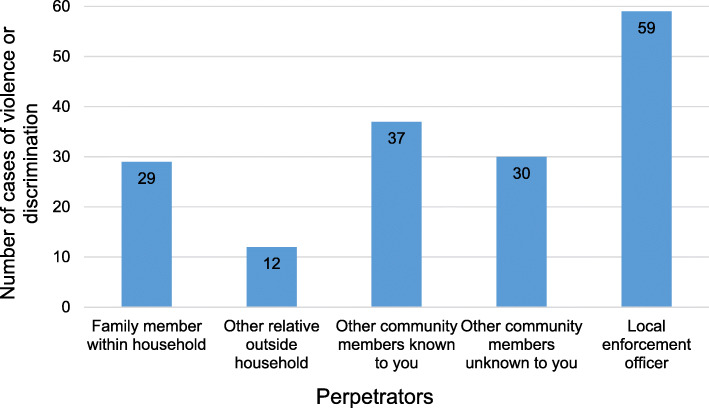
Perpetrators of violence/discrimination, number of cases of violence/ discrimination events (*N* = 167 events), Law enforcement officers most often perpetrated the violence/discrimination (*n* = 59), followed by other community members known to the victim (*n* = 37), other community members unknown to the victim (*n* = 30), family member within household (*n* = 29) and other relative outside the household (*n* = 12)

A total of 167 events of violence/discrimination were reported in our study. Overall, 8.4% (145/1726) of the respondents experienced any form of violence and/or discrimination by any perpetrator during the COVID-19 epidemic in April 2020. The most frequently experienced discrimination was related to one’s social/economic status, reported by 82 (4.8%) participants (Table 2). Of note, 19 (13.1%) of the 145 survivors of violence/discrimination reported more than one perpetrator. Law enforcement officers most often perpetrated the violence/discrimination, as they were incriminated in 59 (35.3%) of the 167 reported violent/discriminatory events (Fig. 1).

**Table 2:** Incidence of violence and discrimination among study participants during the COVID-19 lockdown, April 2020

Characteristic (***N*** = 1726)	Frequency (Percentage)	95% Confidence Interval
**Forms of violence or discrimination reported in Uganda**		
Physical violence at home	21 (1.2)	0.8–1.9
Physical violence outside home	41 (2.4)	1.6–3.2
Discrimination because of my social/economic status	82 (4.8)	3.8–5.9
Discrimination because of my ethnicity, race or nationality	23 (1.3)	0.9–2.9
**Overall experience of different forms of violence or discrimination**		
Yes, only one form	124 (7.2)	6.1–8.5
Yes, more than one form	21 (1.2)	0.8–1.9
Yes, one or more forms	145 (8.4)	7.1–9.8
No violence/discrimination	1581 (91.6)	90.2–92.8

### Factors associated with experiencing violence and/or discrimination among study participants during the COVID-19 lockdown, April 2020

At multivariate analysis, after adjusting for covariates, the odds of experiencing violence and/or discrimination were 1.60 times higher among males compared to females (Adjusted Odds Ratio = 1.60 CI:1.10–2.33). Moreover, having physically attended work for more than 3 days in the past week and inability to access social or essential health services since the epidemic started were also associated with higher odds of experiencing violence and/or discrimination. The value of the Hosmer Lemeshow test statistic for the adjusted model was 0.84 and an associated *p*-value of 0.9744, implying there was no evidence of lack of good fit. Assessment of the ROC curve gave an AUC value of 0.75, showing that the discrimination for the adjusted model was sufficiently good and the reported value of McFadden’s R-squared was 0.054. It can be concluded that the adjusted model had a good fit, demonstrated by an AUC > 0.65 and a Hosmer Lemeshow *p*-value > 0.05 (Table 3).

**Table 3:** Factors associated with experiencing violence and discrimination among Ugandans during the COVID-19 lockdown, April 2020

Variable	Experienced violence or discrimination ***n*** = 145 (%)	Not experienced violence or discrimination ***n*** = 1581 (%) (ref)	Crude OR (95% CI)	Adjusted OR (95% CI)
**Days physically attended at work in the past week**				
0–3 days	101 (69.4)	1249 (79.0)	1.00(ref)	1.00(ref)
> 3 days	44 (30.3)	332 (21.0)	1.64 (1.13–2.3)	1.52 (1.03–2.23)
**Age Group**				
≤ 17 years	2 (1.4)	10 (0.6)	1.00(ref)	
18–28 years	45 (31.0)	400 (25.3)	0.62 (0.13–2.9)	
29–39 years	66 (45.5)	640 (40.5)	0.57 (0.12–2.61)	
40–49 years	20 (13.8)	327 (20.7)	0.34 (0.07–1.62)	
50^+^ years	12 (8.3)	203 (12.8)	0.33 (0.65–1.64)	
**Sex**				
Male	103 (71.0)	912 (57.7)	1.80 (1.24–2.61)	1.60 (1.10–2.33)
Female	42 (29.0)	669 (42.3)	1.00(ref)	1.00(ref)
**Maximum Education**				
Primary & None	2 (1.4)	1 (0.1)	1.00(ref)	
Secondary	7 (4.8)	56 (3.5)	1.00	
Tertiary (certificate, diploma, degree)	86 (59.3)	777 (49.2)	1.87 (0.81–4.31)	
University (masters & Ph.D.)	50 (34.5)	747 (47.3)	1.65 (1.15–2.38)	
**Marital status**				
Single	58 (40.0)	618 (39.1)	1.00(ref)	
Legally married	60 (41.4)	694 (43.9)	1.64 (0.55–4.86)	
Cohabitation	21 (14.3)	226 (14.5)	1.25 (0.28–5.56)	
Divorced & Widowed	6 (4.2)	43 (2.7)	0.99 (0.59–5.66)	
**Currently lives with**				
Parent (s)	27 (18.6)	280 (17.7)	1.10 (0.6–1.75)	
Spouse/partner	65 (44.8)	776 (49.1)	0.91 (0.59–1.40)	
Child (ren)	58 (40.0)	676 (42.8)	0.96 (0.64–1.46)	
Sibling (s) or other relative (s)	36 (24.8)	411 (26.0)	0.98 (0.65–1.47)	
Friends	15 (10.3)	100 (6.3)	1.68 (0.94–3.03)	
Alone	122 (84.1)	1357 (85.8)	1.00(ref)	
**Lives with housemates in age-groups**				
Over 70 years	32 (22.0)	147 (9.0)	1.33 (1.13–1.58)	
Between 18 and 70 years	126 (87.0)	1369 (86.0)	1.00(ref)	
12 to 17 years	67 (46.0)	698 (44.0)	1.04 (0.92–1.18)	
Under 12 years	108 (75)	962 (61.0)	1.22 (1.08–1.37)	
**Lives in**				
Rural/village	27 (18.6)	162 (10.3)	1.00(ref)	
Within Kampala city center	13 (8.9)	173 (10.9)	0.60 (0.35–1.06)	
Kampala suburb	30 (20.7)	658 (41.6)	0.27 (0.16–0.47)	
Other town/city center	44 (30.3)	285 (18.0)	0.45 (0.22–0.90)	
Other suburb	31 (21.4)	303 (19.2)	0.92 (0.55–1.55)	
**Housing conditions**				
House or apartment with garden	44 (30.3)	653 (41.3)	7.40 (0.66–83.4)	
House or apartment No garden	37 (25.5)	436 (27.6)	1.87 (1.15–3.07)	
Apartment with balcony	8 (5.5)	158 (10.0)	3.40 (1.92–6.06)	
Apartment No balcony	29 (20.0)	228 (14.5)	0.75 (0.35–1.63)	
A Room	21 (13.8)	87 (5.5)	1.30 (0.80–1.98)	
Hut, Shack & Homeless	7 (4.9)	16 (1.0)	1.00(ref)	
**What they do for a living**				
Student	23 (15.9)	183 (11.8)	1.12 (0.54–2.34)	
Jobless	11 (7.2)	113 (7.6)	1.00(ref)	
Self-employed	28 (19.3)	256 (16.2)	1.30 (0.59–2.70)	
Work for a person, institution, or company	52 (35.9)	679 (43.0)	0.80 (0.4–1.55)	
Work for the government	31 (21.4)	347 (21.9)	0.92 (0.45–1.89)	
**Current working conditions**				
Worker from home	32 (22.1)	631 (39.9)	0.43 (0.27–0.68)	
Worker in an open space (market, shop, roadside, etc.)	23 (15.9)	95 (6.0)	0.72 (0.39–1.31)	
Worker in a closed indoor space alone (office, etc.)	15 (10.3)	177 (11.2)	0.83 (0.51–1.37)	
Worker in a closed indoor space with several others (office, etc.)	27 (18.6)	273 (17.3)	2.04 (1.18–3.52)	
Not applicable (jobless or student)	48 (33.1)	405 (25.6)	1.00(ref)	
**Wealth Index**				
1st Quintile (poorest)	50 (34.5)	302 (19.1)	1.00(ref)	
2nd Quintile	31 (21.4)	308 (19.5)	0.61 (0.38–0.98)	
3rd Quintile	33 (22.8)	335 (21.2)	0.59 (0.37–0.95)	
4th Quintile	22 (29.0)	459 (29.0)	0.29 (0.17–0.45)	
5th Quintile (richest)	9 (6.2)	177 (11.2)	0.31 (0.15–0.64)	
**Satisfied with staying at home (on a scale of 5)**				
Not Satisfied (1)	23 (15.9)	111 (7.0)	1.00(ref)	
2	23 (15.9)	110 (6.9)	1.10 (0.53–1.90)	
3	35 (24.1)	313 (19.8)	0.54 (0.31–0.95)	
4	28 (19.3)	375 (23.7)	0.36 (0.20–0.65)	
Very Satisfied (5)	36 (24.8)	672 (42.5)	0.26 (0.15–0.45)	
**Has difficulty obtaining food**				
Yes	100 (69.0)	634 (40.1)	3.32 (2.30–4.80)	
No	45 (31.0)	947 (59.9)	1.00(ref)	
**Overall failure to access food and/or essential health services**				
Yes (were not able to access food and/or essential health services)	102 (70.3)	677 (42.8)	3.20 (2.20–4.60)	3.10 (2.14–4.50)
No	43 (29.7)	90.4 (57.2)	1.00(ref)	1.00(ref)

Adjusted model: McFadden R^2^ = 0.054, Hosmer Lemeshow test (Chi2 = 0.84, *p* value = 0.9744), ROC Curve AUC = 0.75

## Discussion

This study assessed the incidence and factors associated with experiencing violence and/or discrimination among Ugandan residents during the initial phase of the COVID-19 lockdown. Overall, 8.4% experienced any of the four forms of violence and discrimination during the one month of the lockdown. To the best of our knowledge, this level of violence and/or discrimination cannot be compared to other studies in Uganda including the UDHS which reports over one year primarily through households while this study reports the incidence of violence and discrimination in only one month [16]. However, this study extends our knowledge of the incidence of community violence and/or discrimination during an epidemic, including by law enforcement as well highlighting being male and the ability to access essential services as influencers of this violence and/or discrimination. Although not assessed in this study, this high level of violence and discrimination could be attributed to stressors including the long stay at home duration, frustration, boredom, inadequate supply of essential goods, and fear of infection due to the epidemic and the control measures with the resultant job and income losses as well as law enforcement encounters [21].

This study reached the higher socio-economic and education participants—half had tertiary education while nearly the remaining half (46.2%) had a post-tertiary level of education, 40.4% lived in a house or apartment with a garden, 59.0% were residing in Kampala city center or suburb, and only 7.2% were unemployed. Thus, the incidence of violence could be an underestimation since studies have shown that wealthy and highly educated individuals are less likely to experience violence and discrimination [22]. Previous research from Sub-Saharan Africa including in Uganda has shown that levels of most forms of violence and discrimination are strongly conditioned by inequalities within populations including the impact of urban versus rural factors. For example, a survey in Rakai district – in rural Uganda, found that 30% of women had experienced physical violence from their current partner, while a survey among women living with HIV/AIDS in eastern Uganda found that rural residence was associated (OR = 4.4, CI: 1.2–16.2) with a higher risk of intimate partner violence [23, 24]. Rural settings, very often are financially constrained, with poor public health systems, some affected by civil wars and conflicts, have less social protection, and other services, hence they are commonly expected to experience greater challenges addressing the health, social and economic impacts of an epidemic including violence and discrimination [25]. Additionally, it has been documented that most unreported cases of most forms of violence and/or discrimination in Uganda are concentrated in rural areas probably due to poor sensitization and awareness of individual’s rights and poor coverage or access to protective services [26].

The COVID-19 lockdown could have worsened these inequalities within urban versus rural communities or households depending on how the control measures were instituted in each setting. However, the COVID-19 pandemic has been largely concentrated in cities and urban areas versus rural settings, including in Uganda where Kampala and Wakiso districts have been the epicenters throughout the epidemic and had more stringent enforcement of the control measures. All in all, from our study findings, the COVID-19 epidemic could have resulted in a trade-off in levels of violence and discrimination between the urban and rural settings, requiring comparative research inclusive of participants from both urban and rural settings.

This high incidence of violence and discrimination from this survey agrees with the patterns reported around the world. In China, it was reported that domestic violence more than tripled during the lockdown in February, and 90% was related to the COVID-19 epidemic [27]. Brazil reported a 40–50% rise in domestic violence and a 30% increase was observed in Cyprus during their COVID-19 lockdowns [27]. Additionally, some countries in Sub-Saharan Africa reported sharp increases in various forms of violence- in Nigeria there was a 149% monthly increase in reports of GBV cases following institution of lockdowns while in the same month, Kenya experienced a spike in reported sexual offenses, constituting 35.8% of the criminal matters reported in early April [4, 9].

Our findings show that law enforcement officers perpetrated more than one-third of the reported violence and discrimination. To ensure compliance with COVID-19 preventive measures, strategies such as curfews, banning of gatherings, and unnecessary movements were backed by heavy deployment of law enforcement officers such as police and local defense forces countrywide. Scuffles between the enforcement officers and the public more especially during the curfew hours were frequently cited in the local media reports during the COVID-19 epidemic [28]. The odds of experiencing any form of violence and discrimination were significantly higher among males compared with females. Experiencing violence and discrimination between males and females is often dependent on the form of violence, the perpetrator, the setting, and the individual’s characteristics. The highest homicide rates worldwide are among males while women are more likely to experience violence at home [29]. Globally, although violence is known to be common in most settings and with victims being overwhelmingly females, instabilities resulting from emergencies such as the COVID-19 lockdown could have disrupted the norm and led to circumstances where men who left their homes were more frequently presented with the law enforcement encounters [30].

It has also been documented that societal and economic pressures play into harmful gender norms in communities disproportionately affecting men and women [31]. Our findings are in line with reports indicating that societal and economic pressures are very often directed towards men than women which can risk men developing or maintaining frustrations and harmful stereotypes such as resorting to violence to somehow uphold the ideal of masculinities which violence can be directed home or even towards other community members [9, 31].

The turbulence resulting from the COVID-19 crisis could have compounded this situation, resulting in more pressure on men to protect and provide for the families, which could explain why more men in this study were affected.

Similarly, individuals who had attended work physically for more than 3 days in the past week were more likely to experience any form of violence and discrimination, and the violence mostly occurred outside of the home, also probably related to violation of the measures. This is in agreement with some reports in early April that cited local enforcement in Uganda had been accused of beating fruits and vegetable sellers and motorcycle taxi riders who had refused to clear the streets, as well as accounts of police violence as far as inside homes in South Africa and Kenyan neighborhoods involving the urban poor who were attempting to continue with work [32, 33].

The odds of experiencing any form of violence and discrimination were higher for those who were unable to access social or essential services compared to those who were able to access them. These findings are in agreement with findings from quarantine experiences during a (Severe Acute Respiratory Syndrome) SARS outbreak in Canada in 2003 which indicated that having inadequate essential supplies such as food, water, clothes, accommodation, and medicines was a major source of agitation and frustration in the communities [34]. The COVID-19 lockdown resulted in unanticipated and prolonged forced co-existence in the Ugandan homes and communities amidst the economic and financial frustration with struggles to access essential services and supplies which could have resulted in encounters with law enforcement during the curfew hours as well as disputes at the household level [35]. While instituting the COVID-19 social control measures, the Ugandan government included a program to distribute food and measures to support those that needed essential health services including seeking permission to move from the local government officials [36]. However, there were continuous media reports of Ugandan communities struggling to transmit sick people to the health facilities and access food throughout the lockdown period [35]. This absence of a pre-existing or coordinated system that ensures continuity of essential services such as food and drugs for those with chronic illnesses during an epidemic crisis could have led to frustrations at both household and community levels as well as increased police-citizen encounters resulting in increased cases of violence and discrimination [35].

The findings in our study are quite different from those documented in research on violence and discrimination in most Sub-Saharan African countries during the HIV/AIDS epidemic. Some of the factors that are commonly cited to increase levels of violence and discrimination while implementing control measures for HIV/AIDS include sexual orientation- commonly towards the Lesbian, Gay, Bisexual, Transgender, and Intersect (LGBTI), females- there has been increased attention over the years towards the linkages between HIV/AIDS and violence against women, younger persons, lack of education, social and cultural practices including wife inheritance, child marriages and polygamy [37–39]. For example, according to a cross-sectional South African study, young women aged 15–26 years who experienced intimate partner violence were 50% more likely to have HIV than young women who had not experienced violence, while the UNAIDS assessment conducted in 2014 revealed that young women in Sub Saharan Africa face higher levels of spousal physical or sexual violence than women from other age groups [39–41]. A multi-country study conducted in Burkina Faso, Kenya, Malawi, and Uganda to evaluate experiences of HIV-related discrimination found that women were more likely to experience interpersonal discrimination than men [42]. This difference in findings can be explained by the nature of the HIV/AIDS epidemic vs the COVID-19 epidemic. Most of the forms of violence and discrimination documented during the HIV/AIDS epidemic are commonly physical, emotional, and sexual from intimate partners and HIV/AIDS status or gender-based [38–40, 42]. On the other hand, violence and discrimination resulting from the social-economic disruptions of the epidemic including community violence predominantly by law enforcement and domestic violence due to the long stay-at-home policies were the most common forms in the COVID-19 epidemic [4].

Similar to our study findings, during the large Ebola outbreak in West Africa (2014–2015), as response efforts focused on containing the disease, there were reports of increased cases of violence and discrimination [43]. Guinea reported a 4.5% increase in sexual and gender-based violence with twice as many rapes while Sierra Leone and Liberia recorded more cases of gender-based violence during the Ebola epidemic [43]. However, these accounts of violence were commonly sexual and gender-based violence against women and girls, and most of the stigmatization was towards the Ebola survivors [43]. Experiences from previous epidemics including HIV/AIDS, Ebola Virus Disease, Zika, and SARS have shown that Public Health Emergencies tend to exacerbate existing health issues or related problems such as violence and discrimination [44]. Before an epidemic crisis such as COVID-19 with complex population-based control measures including lockdowns, high levels of economic hardships/insecurity will make it increasingly difficult for the population to comply with the recommended public health control measures or restrictions, posing huge problems including violence and discrimination [44]. This highlights the need for public health policies and support systems with the social/economic viewpoint of the population accompanied by the integration of concurrent systematic tracking and mitigation of violence and discrimination in disease outbreak response [45].

### Study strengths and limitations

The online approach missed out on individuals without internet access or those who could not afford to pay for the social media tax eventually skewing the study population to the higher socioeconomic respondents, who are less likely to experience violence or discrimination and could potentially underestimate the level of violence and discrimination.

The combination of both violence and discrimination accounts into one dependent variable for analytical purposes could affect the generalization of our study findings to the independent experiences of violence or discrimination.

Additional questions or evaluations were not done to ascertain how our participants had perceived violence and/or discrimination which perceptions could have varied widely. However, the findings still show high levels of violence and discrimination, an important area with limited documentation and that requires attention during the ongoing COVID-19 response. Additionally, we recommend further studies including face to interviews with qualitative approaches to further explore the phenomenon and platforms that can explore both violence and discrimination simultaneously.

## Conclusion

The incidence of violence and discrimination among Ugandan residents during the COVID-19 lockdown was high and mostly perpetrated by law enforcement officers due to the strict lockdown measures. Males, individuals who had attended to work physically for more than 3 days in the past week and those who had difficulties accessing social or essential health services were more likely to experience violence and discrimination.

Institution of lockdowns may be a necessary intervention to reduce the spread of COVID-19 in some situations as the attention is often focused on controlling the epidemic, but they have serious psychological and social disruptive consequences. We recommend that it is important to mitigate the collateral impact of lockdowns where possible with interventions that focus on improving policing quality to reduce law enforcement violence and ensuring continuity of access to essential services as well as strengthening support systems for vulnerable groups including males and persons of low economic status. More research is needed to explore alternative models of ensuring compliance to prevention measures in epidemics including models of risk communication and community mobilization.

## Data Availability

The datasets upon which our findings are based belong to the ICP COVID-19 project. For confidentiality reasons, the datasets are not publicly available. However, the data sets can be availed upon reasonable request from the corresponding author and with permission from the ICP COVID-19 project.

## Abbreviations

COVID-19: Corona Virus Disease 2019
HDREC: Higher Degrees, Research and Ethics Committee
GBV: Gender-Based Violence
ICP: International Citizen Project
MoH: Ministry of Health
SARS: Severe Acute Respiratory Syndrome
UDHS: Uganda Demographic and Health Survey
UNCST: Uganda National Council for Science and Technology
UPHFP: Uganda Public Health Fellowship Program
WHO: World Health Organization
